# Recruiting men from across the socioeconomic spectrum via GP registers and community outreach to a weight management feasibility randomised controlled trial

**DOI:** 10.1186/s12874-020-01136-2

**Published:** 2020-10-06

**Authors:** Matthew D. McDonald, Stephan U. Dombrowski, Rebecca Skinner, Eileen Calveley, Paula Carroll, Andrew Elders, Cindy M. Gray, Mark Grindle, Fiona M. Harris, Claire Jones, Pat Hoddinott, on behalf of the Game of Stones team, Alison Avenell, Frank Kee, Michelle McKinley, Martin Tod, Marjon van der Pol

**Affiliations:** 1 Nursing, Midwifery and Allied Health Professions Research Unit, University of Stirling, Stirling, UK; 2 Physical Activity and Wellbeing Research Group, Curtin University, Perth, Australia; 3 School of Psychology, Curtin University, Perth, Australia; 4 Faculty of Kinesiology, University of New Brunswick, Fredericton, New Brunswick, Canada; 5 Division of Psychology, University of Stirling, Stirling, UK; 6 Men’s Health Forum in Ireland, Dublin, Ireland; 7 Nursing, Midwifery and Allied Health Professions Research Unit, Glasgow Caledonian University, Glasgow, UK; 8 School of Social and Political Sciences, Institute of Health & Wellbeing, University of Glasgow, Glasgow, UK; 9 Institute for Health Research and Innovation, University of the Highlands and Islands, Inverness, UK; 10 Health Informatics Centre, University of Dundee, Dundee, UK

**Keywords:** Recruitment, Randomised feasibility trial, Weight management, Obesity, Health inequalities, Primary care, Community outreach, SMS, Financial incentives

## Abstract

**Background:**

Men, particularly those living in disadvantaged areas, are less likely to participate in weight management programmes than women despite similar levels of excess weight. Little is known about how best to recruit men to weight management interventions. This paper describes patient and public involvement in pre-trial decisions relevant to recruitment and aims to report on recruitment to the subsequent men-only weight management feasibility trial, including the: i) acceptability and feasibility of recruitment; and ii) baseline sample characteristics by recruitment strategy.

**Methods:**

Men with BMI ≥30 kg/m^2^ and/or waist circumference ≥ 40 in. were recruited to the feasibility trial via two strategies; community outreach (venue information stands and word of mouth) and GP letters, targeting disadvantaged areas. Recruitment activities (e.g. letters sent, researcher venue hours) were recorded systematically, and baseline characteristics questionnaire data collated. Qualitative interviews (*n* = 50) were conducted three months post-recruitment. Analyses and reporting followed a complementary mixed methods approach.

**Results:**

105 men were recruited within four months (community *n* = 60, GP letter *n* = 45). Community outreach took 2.3 recruiter hours per participant and GP letters had an opt-in rate of 10.2% (*n* = 90/879). More men were interested than could be accommodated. Most participants (60%) lived in more disadvantaged areas. Compared to community outreach, men recruited via GP letters were older (mean = 57 vs 48 years); more likely to report an obesity-related co-morbidity (87% vs 44%); and less educated (no formal qualifications, 32% vs 10%, degree educated 11% vs 41%). Recruitment strategies were acceptable, a sensitive approach and trusting relationships with recruiters valued, and the *‘catchy’* study name drew attention.

**Conclusions:**

Targeted community outreach and GP letters were acceptable strategies that successfully recruited participants to a men-only weight management feasibility trial. Both strategies engaged men from disadvantaged areas, a typically underserved population. Using two recruitment strategies produced samples with different health risk profiles, which could add value to research where either primary or secondary prevention is of interest. Further work is required to examine how these strategies could be implemented and sustained in practice.

**Trial registration:**

ClinicalTrials.gov: NCT03040518, 2nd February 2017.

## Background

The combined prevalence of overweight and obesity is higher in men than women in the UK [1, 2], but men are less likely to participate in weight management programmes [3–5]. This phenomenon is not exclusive to weight control, with men often underrepresented in health behaviour change interventions [6, 7]. Using gender-sensitised language for health-related communication targeted at men is in line with Men’s Health Forum (a men’s health charity) guidance [8]. However, recent efforts to tailor language and imagery in advertising for mixed-gender physical activity and weight management interventions, to boost participation of men, have been largely unsuccessful [9, 10]. Qualitative evidence suggests that many existing weight management services are viewed by men as incompatible for their needs [11, 12]. To appeal to men more broadly, systematic review evidence suggests that recruitment strategies designed specifically to engage participants in men-only weight management interventions are required [3]. The need for recruitment strategies that build trust and rapport with men, and are congruent with masculine identities, has been documented [13–16].

Targeted recruitment to gender sensitised interventions delivered within sporting contexts have drawn on the appeal of sports clubs to engage men [17–21]. For instance, recruitment strategies employed in the Football Fans in Training (FFIT) weight management randomised controlled trial included advertisements on club/fan websites, in-stadia advertising, engaging supporters’ groups, local and national media coverage, workplace advertising and in-person match-day recruitment drives, with a team of fieldworkers supporting the recruitment of 1080 men across 13 clubs within four months [20]. A community-based physical activity programme targeting inactive men (Men on the Move) also employed a comprehensive range of recruitment strategies, adopting a strengths-based approach based on creating trust, rapport, and meaningful relationships with men [22]. Specific recruitment strategies included text and email invitations via existing databases, website advertising and social media, a local media campaign, General Practice (GP) referral and snowballing, with 927 men recruited within two weeks across 8 counties and 30 host venues [23]. These examples demonstrate the value in using coordinated, well-resourced, gender-sensitive approaches to recruiting men to weight management and physical activity interventions.

In contrast, recent UK and US based men’s weight management studies struggled to meet recruitment targets [24, 25]. Moreover, the socioeconomic distribution of research samples is frequently skewed in favour of the well-educated and advantaged, with recruitment of disadvantaged men particularly challenging [4, 23, 25–28]. For example, whilst two men-only weight-loss trials reported efficacy at 6 months; only one participant (of 65) recruited for the SHED-IT trial in Australia resided in the most disadvantaged quintile area [28], and the sample recruited for the Rethinking Eating and FITness (REFIT) intervention in the US was highly educated (83.2% university educated) [26]. Building on REFIT, the Gutbusters trial sought to recruit less educated men; but after initial poor recruitment rates, altered the inclusion criteria to include men from any educational background, resulting in another highly educated sample (80.4% university educated) [25].

The social construction of masculinities depends on the social context of men’s lives, and intersects with socioeconomic factors [29]. One reason some men from lower socioeconomic circumstances may be less likely to participate in weight management is the preservation of traditional hegemonic masculine traits such as denying vulnerability or weakness, the need to appear strong, resilient or in control, and reluctance to seek medical assistance [30, 31]. Weight loss itself can be viewed as a feminine space [32], and dieting a woman’s domain [33]. Whilst the innovation of gender-sensitised interventions has increased the appeal of these types of interventions to many men, overcoming strong notions of traditional masculinity, particularly amongst those from lower socioeconomic circumstances, remains a challenge.

Interventions not tested for effectiveness across the socioeconomic spectrum may augment inequalities – conflicting with policy highlighting health inequalities as a key priority [34]. Low socioeconomic status is predictive of poor diet, physical inactivity, and increased risk of morbidity and premature mortality [35–38]. For example, the average life expectancy of men from birth in the most deprived decile areas in Scotland is 13 years shorter than men born in the least deprived (69.7 vs 82.7 years), with this difference less pronounced in women (75.7 vs 85.3 years) [39]. Furthermore, the relationship between socioeconomic status and health outcomes is more strongly mediated by poor diet and physical inactivity in men than in women [40]. Men from more disadvantaged areas, are therefore not only the least likely to access support to manage their weight [4, 25–28], but are often the population that would benefit most from support.

Evidence exists for successful recruitment of men from disadvantaged areas in contexts other than weight management. The Texting to Reduce Alcohol Misuse study recruited 825 men from disadvantaged areas through GP registers and community outreach strategies [41]. Previous work by the same authors concluded that recruitment via primary care alone may miss some men from the target group (harmful drinkers) that could benefit from intervention [42]. Use of both recruitment strategies allowed for broad reach, with community outreach inclusive of men that may not have otherwise engaged or be registered on GP lists [41, 42]. These strategies have also shown promise in recruiting men (*n* = 69) with obesity that drink heavily to a text message based study [43].

Successful recruitment strategies for men-only weight management interventions that specifically target men from disadvantaged communities or from across socioeconomic groups are required. In particular, limited evidence exists for recruitment of men to programmes not delivered within sporting or physical activity contexts, such as remotely delivered interventions using technology (e.g. text message). Detailed reporting of recruitment methods, challenges and successes, as well as participant’s perspectives of the strategies employed, is essential to understand how to better engage men living in disadvantaged areas. This paper describes patient and public involvement in pre-trial decisions relevant to recruitment and aims to report on recruitment to the subsequent men-only weight management feasibility trial, including the: i) acceptability and feasibility of recruitment; and ii) baseline sample characteristics by recruitment strategy.

### Patient and Public Involvement in pre-trial decisions

Patient and Public Involvement (PPI) and stakeholder involvement in Game of Stones [44] was in line with recommendations for involvement of target group representatives and key stakeholders at all stages of research from study design to dissemination [45]. Prior to the trial commencing, men with obesity were consulted on matters relevant to recruitment, including the study name selection, development of recruitment materials and the recruitment strategies to be employed.

#### Recruitment materials

**Fig. 1:**
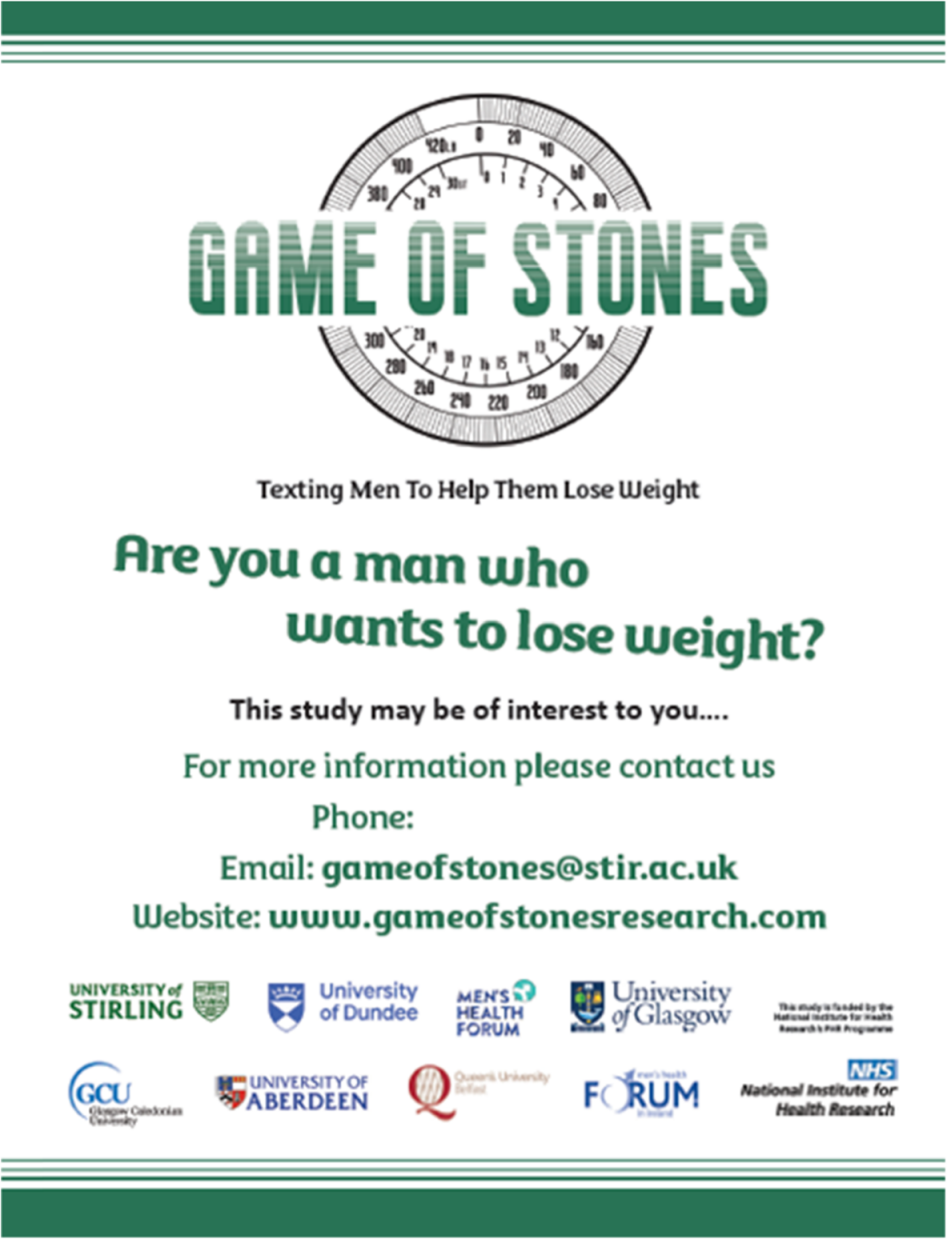
Game of Stones Study Recruitment Poster/Flyer. Should not be reproduced without the permission of the study Principal Investigators: p.m.hoddinott@stir.ac.uk and stephan.dombrowski@unb.ca

Researchers met men with obesity (*n* = 6) on a one-to-one basis to review the study materials (GP invitation letter and study information leaflets) to ensure the language used was appropriate and understandable. These individuals were recruited through researcher contacts including Men’s Shed (www.menssheds.org.uk/) members (*n* = 2), men who had previously taken part in the FFIT programme (n = 2) [20], a former National Health Service (NHS) weight management programme participant (*n* = 1) and a community worker who works with men in disadvantaged areas (n = 1). The researchers took notes during these meetings to capture the feedback gained and, where appropriate, changes to the study materials were made. The phrase *Are you a man who wants to lose weight?* was deemed appropriate by PPI to appeal to men (Fig. 1).

#### Study naming

An initial list of 91 study names was collated from suggestions made by men with obesity, men’s health charities, study co-investigators and University of Stirling staff. The Men’s Health Forum in Ireland then gained PPI email feedback on the list of study name suggestions from 75 men from a range of backgrounds across Ireland including members of; Men’s Sheds; a young men’s project; a lesbian, gay, bisexual and transgender support group; a rural farmers project; a separated father’s support group; sporting clubs, and users of the Men’s Health Forum in Ireland’s online resources. Key considerations for the selection of the study name put forward by these 75 men included to; i) avoid stigmatising language such as the word ‘fat’; ii) avoid words associated with women’s programmes like the word ‘slimmer’; and iii) be cautious about being too smart, for example younger men liked the name suggestion ‘W8M8’, but this suggestion was poorly understood by older respondents. From the initial long list of suggestions, the five most popular study names amongst the 75 respondents were ‘Game of Stones’, ‘Guts 2 Lose’, ‘tXtMEN’, ‘Lose it or Lose Out (LILO)’ and ‘Lean Mean Texting Machine’. Based on the considerations put forward by the men, the latter two suggestions were removed due to some reservations about the use of the word ‘lean’ and the potential negative focus of ‘Lost Out’. The remaining three most popular study names were voted on by attendees at a pre-trial Stakeholder Workshop (*n* = 27, including 8 PPI representatives). The workshop details, which discussed issues besides recruitment, are available elsewhere [39]. ‘Game of Stones’ received the most votes at the Stakeholder Workshop and was selected as the study name.

#### Focus group in trial set-up period

An audio-recorded focus group with men (*n* = 5) living in disadvantaged areas was conducted. The aim of the focus group was to explore men’s views on the planned recruitment strategies and to inform the research team’s approach to selecting venues to recruit men living in disadvantaged areas. Focus group participants were recruited through a researcher community link. Focus group participants were, on average, 52 years old (range 32–58), had an average of 1.8 household members (range 1–3) and four were classified as overweight or obese. All men lived in an area classified by the Scottish Index for Multiple Deprivation (SIMD; area-based index that allocates scores based on income, employment, housing, health, education and access to communication) as living within the most deprived quintile area (SIMD 1). The researcher used a focus group plan and topic guide to steer the discussion and used recruitment materials (e.g. the draft GP participant invitation letter) to support the questions asked. This focus group plan and topic guide is published elsewhere [44].

Key themes raised in the focus group regarding recruitment within disadvantaged areas centred around researcher safety, scepticism of unfamiliar people, the importance of trust and masculinity as a barrier to participation.

#### Community recruitment and safety

Focus group participants warned researchers to be cautious about how they approached men in the community. Some felt that men would deliberately ‘*body swerve’* researchers attempting to recruit from within public spaces, often because they would assume that they are ‘*wanting my debit card’* and that *‘there’s going to be a cost’*. They also expressed concern about researcher safety, joking that one possible outcome was to get ‘your c**t kicked in [Laughter]’. In particular, participants advised that on-street approaches would be unwelcome because they would ‘*think you were from the social [government department responsible for benefits provision]’*. On-street approaches were felt to be particularly ineffective or ‘*dodgy’* within more disadvantaged areas.

‘*It’d probably work some place like in [advantaged area], but in the schemes [disadvantaged areas] you’d just get ridiculed’.*

#### Relationships, trust and familiarity

Focus group participants felt that a lack of relationship with the researcher could be a barrier to the recruitment of men from more disadvantaged areas due to ‘*issues with trust and reassurance*’. They suggested that going to ‘*the heart of communities’* by recruiting within local spaces and community centres and engaging with local, trusted, staff would be the most effective way to build-up relationships. Moreover, masculine identity was viewed as another major barrier to participation, which may be at least partially broken down if recruiting within environments that men are comfortable in.

‘*See you’ve got this [area name], everybody’s a hard man and I’m not doing that’s stuff for wimps and all that, but I think if you get them in their own environment, like i.e. this place [local community centre], it would be a lot better’*

The importance of trust and relationships, as well as listening to and consulting with potential participants was viewed as particularly important; otherwise sceptical community members may just ask; ‘*what’s the con?’*

## Methods

Game of Stones was a three arm men-only weight management feasibility randomised controlled trial with a Short Message Service (SMS) intervention, with and without financial incentives, in comparison to waiting list control for SMS. The possibility (1 in 3 chance) of being randomised into the intervention group that included a financial incentive component was mentioned within the recruitment materials, but to minimise the risk of disappointment bias upon randomisation, no further information on the incentives (e.g. amount of incentive available) were provided during recruitment. Detailed information on interventions, methods and results are published elsewhere [46]. This paper reports secondary mixed-methods data that focuses on trial recruitment only. To assess the feasibility of the recruitment strategies, a target of 105 men within a 4 months period was pre-specified as one of the criteria for progression to a full trial. Men aged 18 or over, with an objectively measured Body Mass Index (BMI) ≥30 kg/m^2^ and/or a waist circumference of ≥40 in. were eligible to participate. Further eligibility criteria are reported elsewhere [44]. Prior research, targeting disadvantaged Scottish men to SMS interventions to support reducing alcohol consumption [41–43], helped inform the community outreach and GP register strategies used in the current study. Participants were recruited by a male and a female researcher (MM and RS) from two Health Boards (healthcare providers responsible for specific geographical areas) in Scotland selected to cover disadvantaged urban, suburban, town and more rural populations. Participants were not actively made aware that disadvantaged areas were being targeted and level of disadvantage was not a study eligibility criterion.

### Community outreach

Researchers targeted free to access venues in more disadvantaged areas (i.e. SIMD 1–2) and liaised with venue managers to negotiate times and locations for recruitment activities. Examples of these contacts included community engagement staff at supermarkets, link workers at community centres and council premises staff (e.g. office facility) responsible for reception areas with frequent passers-by. Community recruitment was conducted via study information stands manned by researchers at supermarkets, council workplaces, hospital foyers, gyms and community centres. When passers-by showed interest, researchers provided an information leaflet, discussed the study, answered questions and asked for interested men’s contact details to confirm or arrange face-to-face appointments. In addition, some word of mouth recruitment occurred via community members who saw the study advertised, researcher community links (e.g. local voluntary organisation staff) and information leaflets placed in venues that men may visit (e.g. gambling shops and barbers).

As this was an individually randomised controlled trial, efforts were made to minimise the risk of contamination between the three trial groups. For example, when recruiting within community venues, researchers attempted to avoid recruiting potentially eligible men that were together and did not recruit from existing men’s groups. Community recruitment activities were undertaken during all hours and days so that a range of men including those that work full-time or part time hours, shifts or nightshifts, and unemployed or retired individuals had the opportunity to participate. A researcher assistant pool of ten postgraduate student and staff recruiters was established so that recruiting researchers could always work in pairs to ensure researcher safety and accommodate concurrent interest at information stands. University of Stirling’s safe working practice guidelines were followed.

### GP register letters

The NHS Research Scotland Primary Care Network (NRSPCN), an organisation that supports primary care research operating in Scotland, provided GP practice list sizes, with demographic data on the proportion of patients registered for each SIMD quintile. From this list, the research team selected practices located within more disadvantaged areas (i.e. SIMD 1 and 2) and NRSPCN invited these practices to participate in the study. Men on the practice lists with a documented BMI of 30 kg/m^2^ or higher were sent a GP practice headed invitation letter and study information leaflet. Interested men then either contacted the research team directly or returned a freepost opt-in reply. When it was not possible to contact men showing interest to arrange a face-to-face appointment, a reminder letter was posted from the research team asking them to get in touch if they were still interested in participating.

#### Quantitative data collection

Researchers systematically recorded all recruitment activities such as the number of GP invitation letters sent, hours spent at community venues, number of information leaflets distributed, and number of interested men’s contact details gained. Following informed consent, participant characteristic information (i.e. age, number of co-morbidities, education level, ethnicity and marital status) were gained through self- report questionnaires, and researcher conducted objective measures (i.e. weight, height and waist circumference) at the assessment visit. Full details of the quantitative data collection procedures are reported elsewhere [44].

#### Qualitative data collection

During the feasibility trial, semi-structured face-to-face interviews were audio-recorded and transcribed with intervention group participants three months post-randomisation (*n* = 50 of 69 randomised; 7–61 min, median = 23 min). Wait-list control participants did not have interim appointments. Some (*n* = 6; 7–18 min, median = 11 min) were interviewed about their experiences, including recruitment, at a 12 months appointment post-randomisation. The two researchers (MM and RS) that led recruitment conducted the interviews at venues and times convenient for participants, after quantitative data collection at scheduled assessments. Topic guides covered several topics relating to study acceptability and recruitment. The qualitative methodology is reported in detail elsewhere [44].

#### Analysis

Quantitative community recruitment data were summarised by site, including venue SIMD quintile, researcher time spent at venues, and the numbers of men participating in each stage of the recruitment process (taking a leaflet, assessment for eligibility and randomisation). GP recruitment data were summarised similarly, including practice SIMD quintile, number of invitations sent, the proportion who opted into the study, and the numbers screened for eligibility and randomised. Descriptive summaries of the baseline characteristics of randomised men were tabulated with the number of non-missing responses, mean and standard deviation reported for continuous variables and numbers with percentages reported for categorical variables. Characteristics were compared by recruitment strategy (community v GP) using two-sample t-tests for continuous variables and chi-squared tests for categorical variables. Analysis was carried out using Stata version 13 (StataCorp LP, Texas, USA).

Qualitative interview verbatim transcripts were entered into QSR NVivo (v12) and analysis was informed by the framework approach [47], with independent coding conducted by four researchers (MM, RS, EC and NG). Charting and visualisation techniques were used in research team meetings to ensure robustness and to facilitate the interpretative analysis that, for the purpose of this paper, focused on experiences of recruitment. Matrix coding queries explored SIMD and recruitment strategy to establish patterns in the data and to identify any disconfirming cases that could provide insights to the analysis. The analysis sought to understand variation in views and experiences. Extracts were labelled with anonymised participant number, recruitment strategy (Community or GP) and participant SIMD postcode area. The quantitative and qualitative results have been synthesised narratively in line with a complementary mixed methods approach [48].

#### Ethical issues

Written consent to take part in the research (including an optional audio-recorded interview) was sought when men attended the baseline appointment prior to randomisation and consent was reaffirmed verbally prior to the interviews. Ethical approval was obtained from the North of Scotland Research Ethics Service (Ref: 16/NS/0120). NHS Research and Development approval was also obtained (Ref: FV974). The feasibility randomised controlled trial that the secondary data analysis in this paper is linked to was registered on ClinicalTrials.gov (Identifier: NCT03040518) on 31st January 2017.

## Results

### Feasibility randomised controlled trial

Feasibility of the recruitment strategies was demonstrated by recruiting and randomising 105 men within four months (between 1st March 2017 and 16th June 2017) by two researchers via community outreach (*n* = 60) and GP practices (*n* = 45). More participants were recruited through community outreach due to the time taken for some GP practices to agree to participate and screen practice lists within the pre-specified 4 months window.

### Community recruitment

In total 87 men in the community showed interest in participating and gave their contact information to researchers, of which 60 (60/87, 69.0%) eligible men were randomised. Researchers spent 97.5 h at information stands in 14 community venues, yielding 42 randomised participants. An average of 2.3 h was spent at information stands per participant randomised, not including preparatory work such as time spent negotiating with venue managers to gain permissions or travel. The remaining ‘word of mouth’ community recruited participants (*n* = 18) heard about the study through friends, family, sports workers and community organisations, or by picking up leaflets left in local venues. More detailed information on word of mouth community recruitment can be found in the full study report [44]. Table 1 summarises community recruitment. Venues were mostly in disadvantaged areas and covered urban, town and more rural areas. The venues in less disadvantaged postcodes were a “Do-It-Yourself” hardware store and a sports centre.

**Table 1:** Community Recruitment

		Community Centres	Retail Outlets	Hospital Foyer	Council Premises	Sports Centre	On street	Word of mouth	Total
Sites/venues	n	4	4	1	2	1	2	n/a	14
Days	n	5	7	5	2	2	2	n/a	23
Researcher hours	n	18.5	28.5	25.5	10	12	3	n/a	97.5
SIMD of venue(s)^a^	Mean	1 (1–1)	2 (1–4)	2	2 (2–2)	5	2.5 (2–3)	n/a	n/a
Leaflets given out	n	63	58	43	29	19	6	n/a	218
Contact information	n	13	17	24	6	7	0	20	87
Did not attend assessment	n	4	4	11	1	1	0	1	22
Attended assessment	n	9	13	13	5	6	0	19	65
Ineligible at assessment	n	2	2	0	0	0	0	1	5
Randomised	n	7	11	13	5	6	0	18	60
Researcher hours/randomised	n	2.6	2.6	2.0	2.0	2.0	^b^	n/a	n/a

^a^Practice locations by Scottish Index of Multiple Deprivation quintile (SIMD; 2016). SIMD 1 represents the most deprived postcode quintile areas and SIMD 5 represents the least deprived postcode quintile areas. ^b^ On street approaches were tested, but no participants were randomised via this route

### GP recruitment

Overall, 33 practices were invited (*n* = 13 in Site A, *n* = 20 in Site B) and five practices (*n* = 4 in Site A, n = 1 in Site B) agreed to participate. Two further GP practices (n = 2 in Site B) expressed an interest in participating towards the end of recruitment but were not required as the study was almost full. Table 2 summarises recruitment via GP practices including the number of study invitation letters sent out, opt ins received, and participants randomised across the participating practices. 10.2% (90/879) of men sent study invitation letters opted in, with 45 subsequently randomised. Opt-in rates varied between practices (4.9–14.6%). Some men opted in after recruitment targets were achieved, and therefore were not included in the study (*n* = 37), whilst others that opted in did not attend an assessment after being invited (*n* = 7) or were ineligible (*n* = 1). Recruitment via GP letters was time consuming to set up (i.e. practices agreeing to participate and screening of practice lists) in relation to the fixed recruitment start date and 4 month recruitment target.

**Table 2:** GP Recruitment

		GP1	GP2	GP3	GP4	GP5	Total
SIMD of practice^a^		2	1	1	2	1	n/a
Letters sent out	N	57	347	187	62	226	879
Opted in	N(%)	4 (7.0)	17 (4.9)	27 (14.4)	9 (14.5)	33 (14.6)	90 (10.2)
Opted in when study full	N	0	3	23	9	2	37
Opted in, invited to assessment	N	4	14	4	0	31	53
Did not attend baseline other reason	N	1	2	0	0	4	7
Attended assessment	N	3	12	4	0	27	46
Ineligible at assessment	N	0	1	0	0	0	1
Randomised	N	3	11	4	0	27	45

^a^Practice locations by Scottish Index for Multiple Deprivation quintile (SIMD; 2016). SIMD 1 represents the most deprived postcode quintile areas and SIMD 5 represents the least deprived quintile areas

### Characteristics by recruitment strategy

Table 3 summarises the characteristics of participants by recruitment strategy. Men recruited via GP letters were on average older (mean = 57.1 years) compared to community recruits (mean = 48.3 years), *p* < 0.01; and more GP recruits reported having at least one co-morbidity (39/45, 86.7%) compared to community recruits (26/59, 44.1%), *p* < 0.01. Men recruited via GP practices were more likely to report high blood pressure (30/45, 66.7%) than community recruits (18/59, 30.5%), *p* < 0.01. Both recruitment strategies yielded participants from across the socioeconomic spectrum, with 29 of 45 (64.4%) GP recruited participants living in more disadvantaged SIMD 1 and 2 areas compared with 33 of 59 (55.9%) community recruits, *p* = 0.38. GP recruits more frequently reported having no formal qualifications (14/44, 31.8%) than community recruits (6/59, 10.2%), and men recruited via GP letters reported less education to degree level (5/44, 11.4%) compared to community recruits (24/59, 40.7%), p < 0.01.

**Table 3:** Sample Characteristics by Recruitment Strategy

		Community recruitment (n = 60)			GP recruitment (n = 45)			Total			***p***-value
Sample characteristics											
Age (years)	N, Mean, SD	57	48.3	13.6	45	57.1	10.8	102	52.2	13.1	< 0.01
Weight (kg)	N, Mean, SD	60	112.7	20.9	45	104.3	13.3	105	109.1	18.4	0.02
Height (cm)	N, Mean, SD	60	176.6	6.6	45	172.8	5.4	105	175.0	6.4	< 0.01
BMI (kg/m^2^)	N, Mean, SD	60	36.2	6.9	45	34.9	4.3	105	35.7	5.9	0.29
≥ 25- < 30	N, n, %	60	7	11.7	45	5	11.1	105	12	11.4	
≥ 30- < 35	N, n, %	60	25	41.2	45	24	53.3	105	49	46.7	
≥ 35- < 40	N, n, %	60	14	23.3	45	8	17.8	105	22	21.0	
≥ 40	N, n, %	60	14	23.3	45	8	17.8	105	22	21.0	
Waist circumference (cm)	N, Mean, SD	60	118.6	13.0	45	114.4	9.4	105	116.8	11.8	0.07
**SIMD deprivation category**											
SIMD 1 (most deprived)	N, n, %	59	23	39.0	45	15	33.3	104	38	36.5	
SIMD 2	N, n, %	59	10	16.9	45	14	31.1	104	24	23.1	
SIMD 3	N, n, %	59	8	13.6	45	4	8.9	104	12	11.5	
SIMD 4	N, n, %	59	8	13.6	45	6	13.3	104	14	13.5	
SIMD 5 (least deprived)	N, n, %	59	10	16.9	45	6	13.3	104	16	15.4	0.38
**Highest educational qualification**											
University Degree Educated (=SVQ5) or higher	N, n, %	59	24	40.7	44	5	11.4	103	29	28.2	
Other Formal Qualifications	N, n, %	59	25	42.4	44	19	43.2	103	44	42.7	
No formal qualifications	N, n, %	59	6	10.2	44	14	31.8	103	20	19.4	
Still studying	N, n, %	59	4	6.8	44	2	4.5	103	6	5.8	
Prefer not to say	N, n, %	59	0	0.0	44	4	9.1	103	4	3.9	< 0.01
**Co-morbidities**											
Arthritis	N, n, %	59	9	15.3	45	13	28.9	104	22	21.2	0.09
Cancer	N, n, %	59	1	1.7	45	2	4.4	104	3	2.9	0.41
Diabetes	N, n, %	59	9	15.3	45	10	22.2	104	19	18.3	0.36
Heart attack	N, n, %	59	4	6.8	45	5	11.1	104	9	8.7	0.44
High BP	N, n, %	59	18	30.5	45	30	66.7	104	48	46.2	< 0.01
Stroke	N, n, %	59	3	5.1	45	3	6.7	104	6	5.8	0.73
One co-morbidity only	N, n, %	59	12	20.3	45	21	46.7	104	33	31.7	< 0.01
One or more co-morbidity	N, n, %	59	26	44.1	45	39	86.7	104	65	62.5	< 0.01
Two or more co-morbidities	N, n, %	59	14	23.7	45	18	40.0	104	32	30.8	0.07
**Ethnic group**											
White	N, n, %	59	53	89.8	45	42	93.3	104	95	91.3	
Non-white	N, n, %	59	5	8.5	45	3	6.7	104	8	7.7	
Prefer not to say	N, n, %	59	1	1.7	45	0	0.0	104	1	1.0	0.64
**Marital status**											
Married or Cohabiting	N, n, %	58	37	63.8	45	35	77.8	103	72	69.9	0.12

Note – recorded demographic information that has an N of < 60 for the community, N of < 45 for GP or N of < 105 in total is due to missing data

### Qualitative interviews

#### Study name

The study name, Game of Stones, was viewed as a source of amusement and intrigue which caught the interest of participants, their families and friends. One participant stated that the study name helped set Game of Stones apart from *‘any other Weight Watchers slimming club’*, with another identifying a *‘laddish undercurrent of stones’* within the study name. For some, the study name acted as an initial *‘hook’* that led to their eventual recruitment.

*Game of Stones it's quite a catchy title so it does draw your attention and you want to see what it's about. (220045, Community Recruit, SIMD 2)*

#### Recruitment tagline

The fact that the information leaflet for Game of Stones targeted men and asked, *‘Are you a man who wants to lose weight?’*, resonated with some participants. The straightforward, matter of fact process of self-identifying as a man who wanted to lose weight appealed.

*But it’s really understated the fact that guys want to lose weight……. So I think I saw that and I was like, yeah, this is me 100 %, don’t really know what it is, as long as you’re not asking me to not eat completely, then I think yeah, 100 %. Yeah, that tagline, it works because I’m a man and you want to lose weight, that was just it, factual. (120017, Community Recruit, SIMD 3).*

Participants therefore felt that both the study name and the direct style of the promotional materials played a role in their recruitment to the study. They viewed Game of Stones as a study specifically for them; *‘a men’s thing’*.

#### Trust and familiarity

Trust and familiarity played a role in the successful recruitment of many participants. Men who received a study invitation letter from their GP believed they had been identified as a potential participant with good reason, since the practice staff knew them and their ailments well.

*The doctor's seeing you and he knows you've got high blood pressure, he knows you've got overweight problems/issues, so really he's channelling you to the right place. (110001, GP Recruit, SIMD 1)*

Receiving the study invitation from a trusted source whom participants had a relationship with, potentially built up over many years, was seen as significant. Endorsement from familiar local health professionals as well as the National Health Service seemed to legitimise and validate the invitation. For many, this seal of approval *‘made a bit of a difference’* and influenced their decision to participate.

*The fact that it came through the GP, I thought kind of legitimised it a little bit, so that’s probably why I said yes to it, gave it a go, and the timing was good because I was looking to do something with this anyway. (110011, GP Recruit, SIMD 2)*

Similarly, participants recruited in the community via word of mouth, felt that information being passed on by familiar and *‘friendly people’*, such as local voluntary organisation staff, provided an element of trust that aided engagement. In contrast, trust needed to be established at first meeting with men recruited through encounters at community stalls. Participants’ views on recruitment often appeared to be shaped by the way they were recruited themselves. For example, positive reflections on being recruited after encountering researchers at stalls within community settings, confirming the pre-trial focus group findings that recruiting from familiar, local venues can put men at ease and help establish trust with the recruiter. Having the opportunity to ask questions about the program face-to-face during community recruitment was also valued.

*…it's just more looking at the person explaining it to me directly then the person who is actually involved in the programme. (220039, Community Recruit, SIMD 1)*

#### Place and context

Some community recruited participants alluded to how crucial the choice of venue may be in recruiting men in a manner congruent with their situation and motivations. For example, the foyer area in a large hospital was viewed as ‘*ideal*’, after all, they are *‘all about your health and wellbeing’*. Similarly, in the context of a gym foyer area;

*I suppose in one sense you're going to catch people who are going to the gym to try and lose weight, but also, you're going to catch people who are already motivated. (120030, Community Recruit, SIMD 2)*

Indeed, if targeting the recruitment of men via the community on a larger scale, careful planning will be required in order to ‘*find the places that they like the most’.*

#### Privacy, embarrassment and a sensitive approach

For some, receiving a physical letter from their GP in the privacy of their own home allowed them to make a pressure-free decision about taking part in the study. Having the opportunity to discuss the study with their partner before making an informed decision was valued.

*I think getting it through the door, and then actually [partners name] was there, we both had a wee chat about it and she said, why not, give it a go. Whether I'd have done that outside and brought the stuff back, I don’t know, but certainly when it was through the door and it was in there, it was through the GP, then, yeah, I think it made a bit of a difference, yeah. (110015, GP Recruit, SIMD 1)*

Often, the GP letter acted as a stimulus for participants to act on their weight that they felt was required.

*I got a letter through the post, and it was, right okay, maybe this is the thing that’s going to spark that motivation to actually do it, rather than just, maybe, think about it…… probably more the post than an email or a text coming through: getting a physical letter delivered through the post… (110024, GP Recruit, SIMD 5)*

Some recruited through community venues suggested that a letter from their GP would have *‘slightly offended’* them or felt it would need to be *‘very subtle’*. Others reflected that they would have ignored the letter or dismissed it as *‘junk mail’*. These views may partly explain why a large proportion (89.8%) of men sent a GP letter did not opt into the study. Equally, some GP recruits suggested that they would have gone out of their way to avoid study information stands within community settings. A participant from a disadvantaged area described how strongly averse he would have been to the idea of approaching a community recruitment stall; *‘Never. I’d never go near it’.* Some suggested, as in the pre-trial focus group, that they would automatically associate a researcher’s presence in public places with individuals canvassing for charities or trying to make sales. For others, approaching a stand advertising a weight loss programme within a public place, was perceived as potentially embarrassing or stigmatising.

*I think even men approaching you, it would be in the back of their mind, it would be I’m advertising I’m overweight. You know, so I’ve not wanted to show myself off as a humpty dumpty. (220015, Community Word of Mouth Recruit, SIMD 4)*

#### Summation

No single recruitment strategy was universally well-liked by participants. However, both methods generated a large amount of interest in the study and engaged men that perhaps just needed to come across an intervention they could identify with to prompt them into participation. As one participant put it;

*I think you’ve got the balance right. If you’re doing through the GP referrals and ad hoc meetings at places. I think you’ve got the balance right there. And it’s... I didn’t find it intrusive. In fact, when I saw you, I came over and I thought, this is…maybe it was the trigger I needed. (220017, Community Recruit, SIMD 3)*

## Discussion

Recruiting 105 men within the target of 4-months to a weight management randomised controlled trial via community outreach and GP practice letters was feasible and acceptable to men from diverse backgrounds. Men from across the socioeconomic spectrum were recruited, and both recruitment strategies engaged men, with no one strategy suiting all. The importance of the study name, gender-sensitised language and tagline as a hook, as well as a sensitive approach, trust and familiarity in recruiting were qualitative themes. Compared to community outreach, more men recruited via GP practice letters had no formal qualifications, were not educated to degree level and had one or more obesity-related co-morbidity.

Interest was generated in this study, with 90 men (37 after the study was full) sent a GP letter opting in (see Table 2) and 87 men (see Table 1) encountering community outreach activities passing on their contact information. Of the interested men that researchers attempted to contact to invite to a baseline assessment (*n* = 140), the majority attended a baseline appointment, were eligible and randomised (*n* = 105). This study was relatively low burden for participants, with a maximum of four scheduled one-to-one researcher contacts over 12 months [46]. Individuals from disadvantaged groups may be less likely to participate in programs where the intervention or research procedures are burdensome [49]. Whilst some interventions targeted at men use physical activity and the appeal of sports settings [22, 50], remotely delivered individual interventions can be effective [51], less burdensome and may be more inclusive of men that do not wish to or are unable to attend groups, participate in physical activity or who are not sports fans.

The Game of Stones sample was more disadvantaged than most men-only weight management interventions [20, 25–28]. Disadvantage is linked to having reduced confidence and being less likely to seek health information [52], hence, recruitment strategies specifically targeting disadvantaged communities are required to recruit socioeconomically diverse samples. Men’s attitudes towards their own weight differs, with some more vulnerable to or aware of potential adverse experiences linked to their weight. Weight stigma primes some men with obesity to worry about others judging their appearance [53]. Being seen to approach a weight management recruitment stand in public may challenge men’s masculine persona, with qualitative data suggesting that some participants would have been embarrassed or put off by this. Trust in recruiters has been recognised as important elsewhere [54], and the invitation letter, coming from their own GP, reassured some of the studies validity. However, whilst a sensitive approach to recruitment is required, even when non-stigmatizing language is adopted (e.g. within GP letters), reference to body weight can provoke emotional reactions [55]. This is corroborated by some community recruits stating they may have taken offence at a GP letter inviting them to a weight management intervention. The Men on the Move physical activity study successfully recruited participants via a suite of community strategies, but concluded that more targeted approaches are required to engage a more disadvantaged population [23].

Targeted GP letter and community outreach strategies used in studies conducted by Crombie and colleagues recruited a high proportion of men from disadvantaged areas [41–43]. In the Texting to Reduce Alcohol Misuse (TRAM) study, GP letters were sent exclusively to men with a home postcode in the most disadvantaged quintile areas; and community venues selected in the most disadvantaged quintile areas [41]. In Game of Stones, whilst GP practices and community venues were targeted based on their postcode being in the two most disadvantaged quintile areas, all eligible GP register men were invited regardless of their home postcode. Varying the level of targeting (e.g. GP letters based on practice or invitee postcode), can help achieve a sample almost exclusively from disadvantaged areas (TRAM; SIMD 1, 636/825, 77.1%), or from across the socioeconomic spectrum with a majority from more disadvantaged areas (Game of Stones; SIMD 1&2, 62/104, 59.6%). Interventions using recruitment strategies that do not consider socioeconomic factors, invariably result in less disadvantaged samples that do not represent the burden of disease attributable to obesity [25–27]. Trials specifically targeting disadvantaged groups, or recruiting diverse samples allowing for assessment of intervention effects across socioeconomic groups, can be termed health equity relevant [56]. Analysis of intervention effectiveness across socioeconomic groups is seldom conducted [57], but may be particularly important for men from disadvantaged areas given inequalities for morbidity and mortality compared to women [39, 40].

The 10.2% GP letter opt in rate (see Table 2) observed in Game of Stones is comparable to other weight management trials recruiting via GP registers [4, 5]. Sex-specific information from the Lighten Up trial was available for one large GP practice, in which 7.4% (48/650) of men and 14.4% (101/700) of women invited participated [58]. Similarly, women were twice as likely to enrol (610/7164, 8.5%) as men (300/6785, 4.4%) when referred to weight-loss programmes via primary care [4]. The latter study also demonstrates that individuals residing in less disadvantaged areas (534/6318; 8.5%) are more likely to take up an offer to participate in weight management than those living in more disadvantaged areas (376/7631; 4.9%) [4]. This suggests that men, including those from disadvantaged areas, may be more likely to respond positively to GP invitations when interventions are targeted specifically for them, corroborating qualitative data indicating that men perceived Game of Stones as being for them, a *‘men’s thing’*.

A recent systematic review suggests that telephone reminders for non-responders to study postal invitations may improve recruitment rates, but also noted the potentially substantial cost and workload of adding a phone call to the recruitment strategy [59]. Prior studies have used telephone reminders for non-responders [41] or used direct phone call invitations [60, 61] to successfully recruit disadvantaged groups. Actively following up on individuals who did not respond to the GP invitation letter could have resulted in a higher opt-in rate in the present study but would have added implications for scale up and sustainability.

Men recruited via GP letters were older and more likely to report having an obesity related co-morbidity than community recruits. Obesity related morbidities increase with age: with such men more likely to be registered at a GP practice, attend appointments, and have a documented BMI than their younger counterparts. High blood pressure, an asymptomatic risk factor in cardiovascular disease development [62], was significantly more prevalent in participants recruited via GP letters than community outreach. The overall average age (52.3 years) observed in Game of Stones is typical for weight management research, with recruitment of younger men challenging [9]. Community outreach may serve an important function in recruiting younger men for primary prevention of obesity related disease, whilst GP letters may be better suited to addressing secondary prevention once an obesity related morbidity has been diagnosed. Men often delay seeking support from health professionals until they are ill [63], with public health interventions that prevent ill-health a UK government policy priority [34].

Five GP sites (of 33 approached) from more disadvantaged areas agreed to invite their patients to participate in this study (with an additional two agreeing after recruitment targets were met), demonstrating sufficient buy-in from primary care providers to recruit men from disadvantaged areas. Primary care buy-in may be enhanced when interventions have previously demonstrated effectiveness within a fully powered RCT. Minimal workload was required from practice staff which may be relevant for future sustainability. It cannot be assumed that GP practice uptake in the present study will be reflected in future upscaling. NRSPCN involvement in engaging GP practices maximised research staff capacity for community outreach activities. However, community outreach activities were resource intensive, with a total of 97.5 researcher hours (see Table 1) spent in venues (over 2 h per participant randomised via this method). Some features of community recruitment, such as linking with community organisation and charity networks, may be more sustainable. Further work is required to establish the sustainability of both GP letter and outreach recruitment strategies.

### Strengths and limitations

Extensive PPI, including men from the target population living in disadvantaged areas, addressed a gap identified in systematic reviews: that strategies for men’s weight management interventions are seldom designed with the target group they intend to recruit [64]. Two researchers with experience of community engagement led on recruitment, systematically recorded relevant information, informed individuals about the study and arranged study appointments, providing continuity. Interviews were conducted with most men attending an assessment at 3 months, allowing for the perspectives of a large and diverse group of participants recruited via both recruitment strategies to be considered. Studies seldom report recruitment strategies for behavioural interventions in detail, yet several have struggled to recruit men, particularly from more disadvantaged areas.

Limitations include the relatively small feasibility trial sample, thus interpretation of recruitment trends observed may not be generalisable. The sample of men recruited were predominantly white (91.4%), although this is more diverse than the population in Scotland [65]. Interviews were conducted with intervention group attendees at 3 months post-randomisation, but control group participants were asked for their views on recruitment 12 months post-recruitment, with the process potentially no longer fresh in their mind. Interview data were collected after completion of questionnaires and weight measures; thus, responses may have been framed by these activities. Participants may have provided socially desirable responses with favourable views on recruitment. The qualitative findings only reflect the views of men who enrolled on the study, limiting learning for expanding reach to men who did not opt into the study after receiving a GP letter or encountering community outreach activities. Thus, participant reflections on recruitment activities that successfully engaged them, are likely to be favourable. Receiving regular text messages will not appeal to all men and further research to explore how the reach of SMS interventions can be extended is required.

Assessment of the cost-effectiveness of the recruitment strategies was beyond the scope of this study. The extent to which the recruitment strategies may be implementable and sustainable in practice has not been established.

## Conclusions

This study demonstrates that postcode area targeted community and GP letter recruitment can engage men from disadvantaged areas, a typically hard to reach demographic. The recruitment strategies used were acceptable to a diverse sample of men, recruited a high proportion of men living in disadvantaged areas and met a pre-specified recruitment rate to inform parameters for a future full trial. No single strategy suited all men, with the use of both strategies having the potential to maximise sample diversity, including for the primary and secondary prevention of obesity-related disease. The mixed methods approach highlights factors that contributed to success, the value of up-front investment in optimising recruitment processes with the target population, and the importance of language and adopting a sensitive approach to recruitment of men for weight management. Recruitment strategy decisions should be evidence based and involve input from target group members. Further work is required to examine how these strategies could be implemented and sustained in practice.

## Supplementary information

**Additional file 1.** Game of Stones Three-month Qualitative Interview Topic Guide.

## Data Availability

The datasets used in the current study can be made available upon reasonable request with the corresponding author (MM) and Chief Investigators of the project (PH and SD). Detailed study information is reported elsewhere [44].

## Abbreviations

BMI: Body Mass Index
FFIT: Football Fans in Training
GP: General Practice
NHS: National Health Service
NRSPCN: NHS Research Scotland Primary Care Network
PPI: Patient and Public Involvement
REFIT: Rethinking Eating and FITness
SIMD: Scottish Index of Multiple Deprivation
SMS: Short Message Service
TRAM: Texting to Reduce Alcohol Misuse
UK: United Kingdom
USA: United States of America

## References

[1] NHS Digital. Statistics on obesity, physical activity and diet, England, 2019. In: Statistics on obesity, physical activity and diet; 2019. https://digital.nhs.uk/data-and-information/publications/statistical/statistics-on-obesity-physical-activity-and-diet/statistics-on-obesity-physical-activity-and-diet-england-2019. Accessed 4 April 2020.

[2] Scottish Government. Obesity indicators, Scotland, 2018. In: An Official Statistics publication for Scotland 2018. https://www.gov.scot/publications/obesity-indicators/. Accessed 4 April 2020.

[3] Robertson C, Avenell A, Boachie C, Stewart F, Archibald D, Douglas F (2016). Should weight loss and maintenance programmes be designed differently for men? A systematic review of long-term randomised controlled trials presenting data for men and women: the ROMEO project. Obes Res Clin Pract.

[4] Ahern AL, Aveyard P, Boyland EJ, Halford JC, Jebb SA (2016). Inequalities in the uptake of weight management interventions in a pragmatic trial: an observational study in primary care. Br J Gen Pract.

[5] Jolly K, Lewis A, Beach J, Denley J, Adab P, Deeks JJ (2011). Comparison of range of commercial or primary care led weight reduction programmes with minimal intervention control for weight loss in obesity: lighten up randomised controlled trial. BMJ..

[6] Rongen A, Robroek SJW, van Lenthe FJ, Burdorf A (2013). Workplace health promotion: a meta-analysis of effectiveness. Am J Prev Med.

[7] Bock C, Jarczok MN, Litaker D (2014). Community-based efforts to promote physical activity: a systematic review of interventions considering mode of delivery, study quality and population subgroups. J Sci Med Sport.

[8] Banks I. The HGV Man manual. Yeovil: Haynes; 2005. https://www.menshealthforum.org.uk/man-manual. Accessed 4 April 2020.

[9] Crane MM, LaRose JG, Espeland MA, Wing RR, Tate DF (2016). Recruitment of young adults for weight gain prevention: randomized comparison of direct mail strategies. Trials..

[10] Ryan J, Lopian L, Le B, Edney S, Van Kessel G, Plotnikoff R (2019). It’s not raining men: a mixed-methods study investigating methods of improving male recruitment to health behaviour research. BMC Public Health.

[11] Archibald D, Douglas F, Hoddinott P, Van Teijlingen E, Stewart F, Robertson C (2015). A qualitative evidence synthesis on the management of male obesity. BMJ Open.

[12] Elliott M, Gillison F, Barnett J (2020). Exploring the influences on men’s engagement with weight loss services: a qualitative study. BMC Public Health.

[13] Hunt K, Gray CM, Maclean A, Smillie S, Bunn C, Wyke S (2014). Do weight management programmes delivered at professional football clubs attract and engage high risk men? A mixed-methods study. BMC Public Health.

[14] Lefkowich M, Richardson N, Robertson S (2017). “If we want to get men in, then we need to ask men what they want”: pathways to effective health programing for men. Am J Mens Health.

[15] Oliffe JL, Bottorff JL, McKenzie MM, Hislop TG, Gerbrandt JS, Oglov V (2011). Prostate cancer support groups, health literacy and consumerism: are community-based volunteers re-defining older men’s health?. Health.

[16] Grace B, Richardson N, Carroll P (2018). “... If You’re Not Part of the Institution You Fall by the Wayside”: Service Providers’ Perspectives on Moving Young Men From Disconnection and Isolation to Connection and Belonging. Am J Mens Health.

[17] Gray C, Brennan G, MacLean A, Mutrie N, Hunt K, Wyke S. Can professional rugby clubs attract English male rugby supporters to a healthy lifestyle programme: the Rugby Fans in Training (RuFIT) study 2013–14: Cindy Gray. Eur J Public Health. 2014;24(suppl_2).

[18] Maddison R, Hargreaves EA, Wyke S, Gray CM, Hunt K, Heke JI (2019). Rugby fans in training New Zealand (RUFIT-NZ): a pilot randomized controlled trial of a healthy lifestyle program for overweight men delivered through professional rugby clubs in New Zealand. BMC Public Health.

[19] Wyke S, Bunn C, Andersen E, Silva MN, Van Nassau F, McSkimming P (2019). The effect of a programme to improve men’s sedentary time and physical activity: the European fans in training (EuroFIT) randomised controlled trial. PLoS Med.

[20] Wyke S, Hunt K, Gray C, Fenwick E, Bunn C, Donnan P, et al. Football fans in training (FFIT): a randomised controlled trial of a gender-sensitised weight loss and healthy living programme for men. Public Health Res. 2015; https://www.ncbi.nlm.nih.gov/books/NBK273998/ Accessed 4 April 2020. 25654156

[21] Quested E, Kwasnicka D, Thøgersen-Ntoumani C, Gucciardi DF, Kerr DA, Hunt K (2018). Protocol for a gender-sensitised weight loss and healthy living programme for overweight and obese men delivered in Australian football league settings (Aussie-FIT): a feasibility and pilot randomised controlled trial. BMJ Open.

[22] Carroll P, Harrison M, Richardson N, Robertson S, Keohane A, Kelly L (2018). Evaluation of a gender-sensitive physical activity programme for inactive men in Ireland: protocol paper for a pragmatic controlled trial. J Phys Act Res.

[23] Kelly L, Harrison M, Richardson N, Carroll P, Robertson S, Keohane A (2018). Reaching beyond the ‘worried well’: pre-adoption characteristics of participants in ‘men on the move’, a community-based physical activity programme. J Public Health.

[24] Griffin T, Sun Y, Sidhu M, Adab P, Burgess A, Collins C (2019). Healthy dads, healthy kids UK, a weight management programme for fathers: feasibility RCT. BMJ Open.

[25] Rounds T, Harvey J (2019). Enrollment challenges: recruiting men to weight loss interventions. Am J Mens Health.

[26] Crane MM, Lutes LD, Ward DS, Bowling JM, Tate DF (2015). A randomized trial testing the efficacy of a novel approach to weight loss among men with overweight and obesity. Obesity..

[27] Morgan PJ, Collins CE, Plotnikoff RC, Callister R, Burrows T, Fletcher R (2014). The ‘healthy dads, healthy kids’ community randomized controlled trial: a community-based healthy lifestyle program for fathers and their children. Prev Med.

[28] Morgan PJ, Lubans DR, Collins CE, Warren JM, Callister R (2011). 12-month outcomes and process evaluation of the SHED-IT RCT: an internet-based weight loss program targeting men. Obesity..

[29] Evans J, Frank B, Oliffe JL, Gregory D (2011). Health, illness, men and masculinities (HIMM): a theoretical framework for understanding men and their health. J Men's Health.

[30] Dolan A (2011). ‘You can’t ask for a Dubonnet and lemonade!’: Working class masculinity and men’s health practices. Sociol Health Illness.

[31] Taylor Smith A, Dumas A (2019). Class-based masculinity, cardiovascular health and rehabilitation. Sociol Health & Illn..

[32] Sabinsky MS, Toft U, Raben A, Holm L (2007). Overweight men's motivations and perceived barriers towards weight loss. Eur J Clin Nutr.

[33] Gough B, Flanders G. Celebrating" Obese" Bodies: Gay" Bears" Talk about Weight, Body Image and Health. Int J Men's Health. 2009;8(3):235-53.

[34] Department of Health and Social Care. Prevention is better than cure: our vision to help you live well for longer, London, 2018. In: Office for National Statistics 2018. https://assets.publishing.service.gov.uk/government/uploads/system/uploads/attachment_data/file/753688/Prevention_is_better_than_cure_5-11.pdf. Accessed 4 April 2020.

[35] Drewnowski A, Specter SE (2004). Poverty and obesity: the role of energy density and energy costs. Am J Clin Nutr.

[36] Stamatakis E, Bajekal M, Osborne V, Yar M, Meltzer M (2006). Obesity, eating and physical activity. Focus on health London.

[37] Department of Health and Social Care. Statistics OfN. Health state life expectancies by national deprivation deciles, England and Wales: 2015 to 2017. In: Office for National Statistics 2019. https://www.ons.gov.uk/peoplepopulationandcommunity/healthandsocialcare/healthinequalities/bulletins/healthstatelifeexpectanciesbyindexofmultipledeprivationimd/2016to2018. Accessed 23 September 2020.

[38] Steel N, Ford JA, Newton JN, Davis ACJ, Vos T, Naghavi M (2018). Changes in health in the countries of the UK and 150 English local authority areas 1990–2016: a systematic analysis for the global burden of disease study 2016. Lancet.

[39] Scottish Government. Healthy Life Expectancy in Scottish Areas 2015–2017 In: National Records of Scotland, 2019. https://www.nrscotland.gov.uk/files//statistics/healthy-life-expectancy/15-17/healthy-le-15-17-pub.pdf. Accessed 4 April 2020.

[40] Whitley E, Batty GD, Hunt K, Popham F, Benzeval M (2013). The role of health behaviours across the life course in the socioeconomic patterning of all-cause mortality: the west of Scotland twenty-07 prospective cohort study. Ann Behav Med.

[41] Crombie IK, Irvine L, Williams B, Sniehotta FF, Petrie D, Jones C (2018). Texting to reduce alcohol misuse (TRAM): main findings from a randomized controlled trial of a text message intervention to reduce binge drinking among disadvantaged men. Addiction..

[42] Crombie IK, Falconer DW, Irvine L, Norrie J, Williams B, Slane PW (2013). Risky single-occasion drinking and disadvantaged men: will recruitment through primary care miss hazardous drinkers?. Alcohol Clin Exp Res.

[43] Irvine L, Crombie IK, Cunningham KB, Williams B, Sniehotta FF, Norrie J (2017). Modifying alcohol consumption to reduce obesity: a randomized controlled feasibility study of a complex community-based intervention for men. Alcohol Alcohol.

[44] Dombrowski SU, McDonald M, Van Der Pol M, Grindle M, Avenell A, Carroll P, et al. Text messaging and financial incentives to encourage weight loss in men with obesity: the game of stones feasibility RCT. Public Health Res. 2020. 32902933

[45] Hoddinott P, Pollock A, O’Cathain A, Boyer I, Taylor J, MacConald C, et al. How to incorporate patient and public perspectives into the design and conduct of research. F1000 Res. 2018;7(752).. 10.12688/f1000research.15162.1 PMC6192439 30364075

[46] Dombrowski SU, McDonald M, Van Der Pol M, Grindle M, Avenell A, Carroll P (2020). Game of stones: feasibility randomised controlled trial of how to engage men with obesity in text message and incentive interventions for weight loss. BMJ Open.

[47] The Qualitative Researcher's Companion. 2002 2019/10/05. Thousand Oaks, California: SAGE Publications, Inc. Available from: https://methods.sagepub.com/book/the-qualitative-researchers-companion. Accessed 4 Apr 2020.

[48] O’Cathain A, Murphy E, Nicholl J (2010). Three techniques for integrating data in mixed methods studies. BMJ..

[49] Kelly L, Harrison M, Richardson N, Carroll P, Robertson S, Keohane A (2019). The impact of a gender-specific physical activity intervention on the fitness and fatness profile of men in Ireland. Eur J Pub Health.

[50] Hunt K, Wyke S, Gray CM, Anderson AS, Brady A, Bunn C (2014). A gender-sensitised weight loss and healthy living programme for overweight and obese men delivered by Scottish premier league football clubs (FFIT): a pragmatic randomised controlled trial. Lancet.

[51] Skinner R, Gonet V, Currie S, Hoddinott P, Dombrowski SU (2020). A systematic review with meta-analyses of text message-delivered behaviour change interventions for weight loss and weight loss maintenance. Obes Rev.

[52] Richardson A, Allen JA, Xiao H, Vallone D (2012). Effects of race/ethnicity and socioeconomic status on health information-seeking, confidence, and trust. J Health Care Poor Underserved.

[53] Lozano L, McKenna J, Carless D, Pringle A, Sparkes A. ‘Sorry mate, you’re probably a bit too fat to be able to do any of this’: Men’s Experiences of Weight Stigma and Its Implications. Int J Men's Health. 2016;15(1): 4-23.

[54] Strömmer S, Lawrence W, Rose T, Vogel C, Watson D, Bottell JN (2018). Improving recruitment to clinical trials during pregnancy: a mixed methods investigation. Soc Sci Med.

[55] Puhl R, Peterson J, Luedicke J (2013). Motivating or stigmatizing? Public perceptions of weight-related language used by health providers. Int J Obes.

[56] Jull J, Whitehead M, Petticrew M, Kristjansson E, Gough D, Petkovic J (2017). When is a randomised controlled trial health equity relevant? Development and validation of a conceptual framework. BMJ Open.

[57] McGill R, Anwar E, Orton L, Bromley H, Lloyd-Williams F, O’Flaherty M (2015). Are interventions to promote healthy eating equally effective for all? Systematic review of socioeconomic inequalities in impact. BMC Public Health.

[58] Sharman C LA, Beach J, Denley J, Daley A, Jolly K. Equity of uptake to a free weight loss programme (lighten up) in South Birmingham PCT. Royal College of General Practitioners (Midland Faculty): Annual Faculty Research Meeting. 2009..

[59] Treweek S, Pitkethly M, Cook J, Fraser C, Mitchell E, Sullivan F, et al. Strategies to improve recruitment to randomised trials. Cochrane Database Syst Rev. 2018;2. MR000013. 10.1002/14651858.MR000013.pub6 PMC7078793 29468635

[60] Bennett GG, Warner ET, Glasgow RE, Askew S, Goldman J, Ritzwoller DP (2012). Obesity treatment for socioeconomically disadvantaged patients in primary care practice. Arch Intern Med.

[61] Warner ET, Glasgow RE, Emmons KM, Bennett GG, Askew S, Rosner B (2013). Recruitment and retention of participants in a pragmatic randomized intervention trial at three community health clinics: results and lessons learned. BMC Public Health.

[62] Kannel WB (1975). Role of blood pressure in cardiovascular disease: the Framingham study. Angiology..

[63] Galdas PM, Cheater F, Marshall P (2005). Men and health help-seeking behaviour: literature review. J Adv Nurs.

[64] Robertson C, Archibald D, Avenell A, Douglas F, Hoddinott P, van Teijlingen E, et al. Systematic reviews of and integrated report on the quantitative, qualitative and economic evidence base for the management of obesity in men. Health Technol Assess. 2014;18(35):1-424. 10.3310/hta18350 PMC4781190 24857516

[65] Scotish Government. 2011 Census: key results on population, ethnicity, identity, language, religion, health, housing and accommodation in Scotland - release 2A in: statistics N, editor. National Records of Scotland 2013. https://www.nrscotland.gov.uk/news/2013/census-2011-release-2a. Accessed 4 April 2020.

