# Supplementary material for: Recruiting men from across the socioeconomic spectrum via GP registers and community outreach to a weight management feasibility randomised controlled trial
Source: BMC Med Res Methodol. 2020 Oct 6;20:249. doi: 10.1186/s12874-020-01136-2 (PMC7542377; doi:10.1186/s12874-020-01136-2)
Supplement: Supplementary file 1 — Additional file 1. Game of Stones Three-month Qualitative Interview Topic Guide. [file 12874_2020_1136_MOESM1_ESM.docx]

Game of Stones Three-month Qualitative Interview Topic Guide

1. How has it been (trying to lose weight/keep weight off/not regain weight in this study)?
2. How you think the study has impacted your
   1. Thoughts and feelings towards weight loss?
   2. Behaviours surrounding weight loss? Strategies? What have you tried? What seems to be working for you? What doesn’t seem to be working for you? Is anyone helping/not helping you? Social Networks? Have you talked to anyone about being in this study, if so what were their reactions? What about family/friends/work colleagues – have they commented? Have they helped or hindered in any way?
3. Did you change anything after I saw you 3 months ago? Did the text messages help?
4. What was it like getting the text messages? What parts of the study have you found helpful or unhelpful in your efforts to lose weight? The web pages? The information links on the website? The pedometer? The incentives? The weight loss targets?
5. Is there anything about the study that could be improved (e.g. initial approach, first appointment, information, randomisation, the texts, the website, the incentives, the communication with the research team) if we do it again?
6. Do you have any suggestions for us as researchers/ what would make a difference?
7. Has there been anything that has surprised you about being part of this study? Has there been anything that has upset you or that you have found difficult?
8. Have you met any other men who are involved in this study? Did you discuss the study? Can you tell me a bit about your conversations?
9. Have you seen any information about this study other than what we have given you?
10. When we design research studies about weight loss in men, we are keen to ask questions and measure the things that really matter to the men taking part [Discuss particular items in the questionnaire and seek their views]?
11. It is now possible to link anonymous computer records about your health with anonymous results of Government funded research like the results of this study. Participants in the research are being asked to consent to this. For example, for men in this study who have lost weight, we could find out in future whether they have less diabetes or cancer than men in the study who did not lose weight. This can help researchers and NHS make decisions about whether new initiatives like those in this study can improve health and are good value for money. What are your views about this?
12. How do you feel about the way you were recruited? What other ways could we recruit men to take part? What is likely to encourage men like you to take part. What is likely to put men off? Do you think you would have signed up if you saw a Game of Stones Stall in a supermarket/ receive a letter from your GP/other routes….(besides the one they responded to) ?
13. I am meeting you today in X - what other venues would you like to attend? We want men to stay in the study and come and be weighed over12 months - how do you suggest we keep men in the study?
